# *Listeria monocytogenes* from Food Products and Food Associated Environments: Antimicrobial Resistance, Genetic Clustering and Biofilm Insights

**DOI:** 10.3390/antibiotics13050447

**Published:** 2024-05-14

**Authors:** Adriana Silva, Vanessa Silva, João Paulo Gomes, Anabela Coelho, Rita Batista, Cristina Saraiva, Alexandra Esteves, Ângela Martins, Diogo Contente, Lara Diaz-Formoso, Luis M. Cintas, Gilberto Igrejas, Vítor Borges, Patrícia Poeta

**Affiliations:** 1 Microbiology and Antibiotic Resistance Team (MicroART), Department of Veterinary Sciences, University of Trás-os-Montes and Alto Douro (UTAD), 5000-801 Vila Real, Portugal; adrianaa.silva95@gmail.com (A.S.);; 2 Associated Laboratory for Green Chemistry (LAQV-REQUIMTE), University NOVA School of Science and Technology, 2829-516 Caparica, Portugal; 3 Department of Genetics and Biotechnology, University of Trás-os-Montes and Alto Douro (UTAD), 5000-801 Vila Real, Portugal; 4 Functional Genomics and Proteomics Unit, University of Trás-os-Montes and Alto Douro (UTAD), 5000-801 Vila Real, Portugal; 5 Genomics and Bioinformatics Unit, Department of Infectious Diseases, National Institute of Health Doutor Ricardo Jorge (INSA), Avenida Padre Cruz, 1649-016 Lisbon, Portugal; 6 Animal and Veterinary Research Centre (CECAV), Faculty of Veterinary Medicine, Lusófona University, 1749-024 Lisbon, Portugal; 7 Food Microbiology Laboratory, Food and Nutrition Department, National Institute of Health Doutor Ricardo Jorge (INSA), Avenida Padre Cruz, 1649-016 Lisbon, Portugal; 8 Department of Veterinary Sciences, School of Agricultural and Veterinary Sciences, University of Trás-os-Montes and Alto Douro (UTAD), 5000-801 Vila Real, Portugal; 9 CECAV—Veterinary and Animal Research Centre, University of Trás-os-Montes and Alto Douro (UTAD), 5000-801 Vila Real, Portugal; 10 Associate Laboratory for Animal and Veterinary Sciences (AL4AnimalS), University of Trás-os-Montes and Alto Douro (UTAD), 5000-801 Vila Real, Portugal; 11 Grupo de Seguridad y Calidad de los Alimentos por Bacterias Lácticas, Bacteriocinas y Probióticos (SEGABALBP), Sección Departamental de Nutrición y Ciencia de los Alimentos, Facultad de Veterinaria, Universidad Complutense de Madrid, 28040 Madrid, Spainlcintas@vet.ucm.es (L.M.C.)

**Keywords:** *Listeria monocytogenes*, food safety, antimicrobial resistance, genetic diversity, biofilm formation

## Abstract

*Listeria monocytogenes*, a foodborne pathogen, exhibits high adaptability to adverse environmental conditions and is common in the food industry, especially in ready-to-eat foods. *L. monocytogenes* strains pose food safety challenges due to their ability to form biofilms, increased resistance to disinfectants, and long-term persistence in the environment. The aim of this study was to evaluate the presence and genetic diversity of *L. monocytogenes* in food and related environmental products collected from 2014 to 2022 and assess antibiotic susceptibility and biofilm formation abilities. *L. monocytogenes* was identified in 13 out of the 227 (6%) of samples, 7 from food products (meat preparation, cheeses, and raw milk) and 6 from food-processing environments (slaughterhouse-floor and catering establishments). All isolates exhibited high biofilm-forming capacity and antibiotic susceptibility testing showed resistance to several classes of antibiotics, especially trimethoprim-sulfamethoxazole and erythromycin. Genotyping and core-genome clustering identified eight sequence types and a cluster of three very closely related ST3 isolates (all from food), suggesting a common contamination source. Whole-genome sequencing (WGS) analysis revealed resistance genes conferring resistance to fosfomycin (*fosX*), lincosamides (*lin*), fluoroquinolones (*norB*), and tetracycline (*tetM*). In addition, the *qacJ* gene was also detected, conferring resistance to disinfecting agents and antiseptics. Virulence gene profiling revealed the presence of 92 associated genes associated with pathogenicity, adherence, and persistence. These findings underscore the presence of *L. monocytogenes* strains in food products and food-associated environments, demonstrating a high virulence of these strains associated with resistance genes to antibiotics, but also to disinfectants and antiseptics. Moreover, they emphasize the need for continuous surveillance, effective risk assessment, and rigorous control measures to minimize the public health risks associated to severe infections, particularly listeriosis outbreaks. A better understanding of the complex dynamics of pathogens in food products and their associated environments can help improve overall food safety and develop more effective strategies to prevent severe health consequences and economic losses.

## 1. Introduction

Over the past decade, public health has increasingly focused on foodborne diseases, in which microbiologically contaminated foods play an important role [1]. Among foodborne pathogens, bacteria are the most common ones, causing many diseases in humans and animals. Common bacteria found in foods include *Salmonella species*, *Shigella species*, *Listeria monocytogenes*, *Bacillus species*, *Yersinia species*, *Campylobacter species*, *Clostridium botulinum*, *Clostridium perfringens*, *Escherichia coli*, *Staphylococcus aureus*, and *Vibrio cholera* [2,3].

*Listeria monocytogenes* is a foodborne pathogen isolated from food and food processing environments that is known for its high adaptability to adverse environmental conditions [4] such as low temperature, low pH, high pressure, and high salt concentrations [5] and for being widespread in the environment, including water, soil, and wastewater [6]. This adaptability not only allows for *L. monocytogenes* to grow in a variety of environmental conditions, but also contributes to antimicrobial resistance and biofilm formation on a wide range of surfaces found in food production environments, allowing for *L. monocytogenes* to survive and proliferate [7,8,9]. In the food processing context, incorrect handling during production, processing, storage, and transportation can cause food-borne illness [10], indicating the possibility of *L. monocytogenes* contamination of foods during and after processing [11]. 

*L. monocytogenes*, an opportunistic human pathogen, is a significant public health problem as it is associated with frequent hospitalizations and widespread outbreaks of foodborne illness [12,13]. This pathogen is responsible for the spread of listeriosis, primarily through the ingestion of contaminated food. The disease can cause serious complications such as meningitis and septicemia [10] and is associated with a high morbidity and mortality [14], especially among populations at risk such as the elderly, pregnant women, newborns, and immunocompromised people [4,7]. Although the incidence of listeriosis is relatively low, mortality rate is high, affecting approximately 20–25% of patients [15,16].

The food production environment promotes bacterial development by providing continuous source of nutrients [6]. Pathogens have properties such as the ability to form and persist in biofilms, making it difficult to manage the contamination situation in food production facilities [6,17,18]. *L. monocytogenes* is prevalent in the food industry, particularly in ready-to-eat (RTE) foods, such as in milk [19], cheese [20], smoked fish, ice cream, patés, and vegetables [21]. This is due to its ability to colonize food processing environments through biofilm formation, resistance to sanitizing chemicals, and high tolerance to adverse conditions [17,22,23]. Antimicrobial resistance in microorganisms isolated from various foods is also a major concern. Antibiotics such as aminopenicillin (ampicillin or amoxicillin), benzylpenicillin (penicillin G and gentamicin, often used in combination with aminoglycosides), trimethoprim alone or in combination with sulfamethoxazole, erythromycin, and tetracyclines can treat *L. monocytogenes* infections [22]. *L. monocytogenes* was found to have low activity against cephalosporins, fosfomycin, and macrolides [17,22]. 

In recent years, whole-genome sequencing (WGS) has become increasingly important in determining the characteristics and relationships of diverse *L. monocytogenes* strains isolated in many food productions [1,14] and in linking them to human disease. Bacterial WGS technology is used to accurately identify pathogens and genotypes through techniques such as multiloccus sequence typing (MLST), clonal complex (CC) identification, core genome MLST (cgMLST9, CRISPR-Cas), and serogrouping. It can also detect genetic determinants of antimicrobial resistance, virulence genes, plasmids and mobile genetic elements (MGE), and provide other relevant data to improve our understanding of the pathogen epidemiology, ecology, and evolution [24,25]. High-resolution clustering improves linking cases to detect specific outbreaks, builds confidence in identifying their microbial source, and detect antimicrobial resistance reservoirs. Nowadays, WGS has evolved to the point of common use in research and reference laboratories handling *L. monocytogenes*, allowing real-time routine genomic surveillance and outbreak investigation [24,25,26]. 

The aim of this study was to perform phenotypic and genomic characterization, as well as evaluate the antimicrobial resistance and properties of *L. monocytogenes* strains obtained from food products and food-associated processing environments. This work provides important information of genetic diversity and illustrates the utility of WGS in improving food safety management by establishing relationships between strains and highlights the critical need for improved detection and control methods by directly linking the high morbidity and mortality rates of listeriosis to the imperative for enhanced safety measures in food management. 

## 2. Results

### 2.1. Prevalence and L. monocytogenes Identification

In this present study, 227 samples obtained from food products or food-processing environments were collected and analyzed for the presence of *L. monocytogenes*. All the presumptive *L. monocytogenes* isolates obtained using Oxford medium appeared as gray-to-black-color colonies surrounded by a black halo. Green-to-blue colonies were observed in CHROM agar. A series of biochemical tests, catalase reaction, Gram-stain, hemolysis assessment, and carbohydrate were performed to confirm and differentiate *L. monocytogenes*. The tests confirmed the presence of *L. monocytogenes* through various tests such as Gram-stain, catalase reaction, and hemolysis test. The bacteria appeared as small rods with rounded ends and showed characteristics like narrow β-hemolysis, positive rhamnose fermentation, and negative mannitol and xylose fermentation. The overall positivity of *L. monocytogenes* was 6% (13/227), similarly distributed among food products (*n* = 7) and food-associated environments (*n* = 6). Taxonomic identification of the 13 presumptive *L. monocytogenes* isolates was confirmed by DNA sequencing of the PCR-amplified 16S rDNA (99.8–100% identity).

### 2.2. Antibiogram of L. monocytogenes Isolates from Food Products and Food Associated Environments

Table 1 summarizes the antimicrobial susceptibility profiles of the 13 *L. monocytogenes* from food products and food-associated environments. Resistance to 12 antimicrobial agents, belonging to β-lactams (meropenem and ampicillin), quinolones (ciprofloxacin), lincosamides (clindamycin), macrolides (erythromycin), aminoglycosides (kanamycin and gentamicin), penicillins (penicillin G), rifampicins, glycopeptides (vancomycin), sulfonamides (trimethroprim-sulphamethoxazol), and oxazolidinones (linezolin) was observed. Among the *L. monocytogenes* strains tested, the highest level of resistance observed was for trimethoprim–sulfamethoxazole, with 38.4% (5/13) of isolates showing resistance, followed by erythromycin, of which 15.5% (2/13) isolates showed antimicrobial resistance. Particularly, 7.6% (one isolate) exhibited a multidrug resistance phenotype, indicating resistance to at least three distinct classes of antibiotics. 

### 2.3. L. monocytogenes Typing and Core-Genome Clustering Analysis

**Figure 1:**
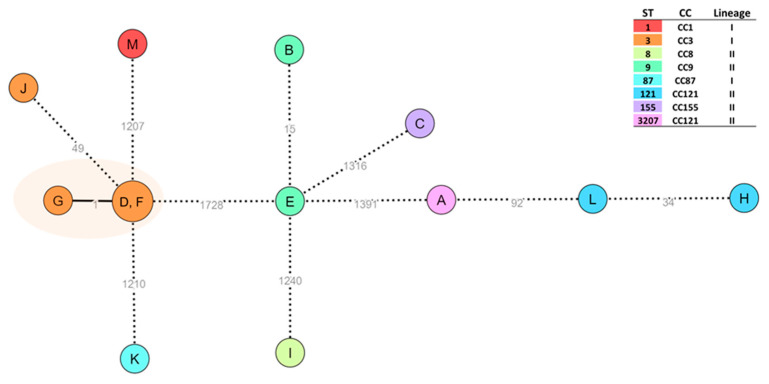
Core-genome clustering analysis of *L. monocytogenes* (thirteen isolates). The Minimum Spanning Tree (MST) was constructed based on the cgMLST 1748-loci Pasteur schema [27]. Each circle (node) contains the sample code and represents a unique allelic profile, with numbers on the connecting lines representing allelic distances (ADs) between nodes. Cluster analysis was conducted with ReporTree v.2.0.3 [28] and data visualization was adapted from the GrapeTree (MSTreeV2 method) dashboard [29]. Straight and dotted lines reflect nodes linked with allelic distances (ADs) below and above a threshold of seven ADs, which can provide a proxy to the identification of genetic clusters with potential epidemiological concordance [30]. Nodes are colored according to the sequence type (ST), with clonal complex (CC) and lineage also presented. The surrounding orange shadow highlights a cluster supported by ≤7 ADs.

Among the 13 *L. monocytogenes* isolates, eight sequence types (ST) were identified: ST1 (*n* = 1), ST3 (*n* = 4), ST8 (*n* = 1), ST9 (*n* = 2), ST87 (*n* = 1), ST121 (*n* = 2), ST155 (*n* = 1), and ST3207 (*n* = 1). A core-genome clustering analysis by cgMLST was performed. The results revealed one genetic cluster of three high closely related ST3 isolates (D, F, and G), with a maximum of one AD between them (Figure 1).

### 2.4. Whole-Genome Sequencing (Antimicrobial Resistance and Virulence Genes)

The genome assembly were characterized by an average sequencing depth of 103.9x, with the number of contigs ranging from 15 to 41 and genome sizes between 2,881,182 and 3,153,118 nucleotides. The complete draft genome sequences of the 13 L. monocytogenes isolates analyzed in this study are deposited in the European Nucleotide Archive (ENA) database under the ENAProject number PRJEB31216. The individual accession numbers for these sequences range from ERS18961302 to ERS18961314.

Among the 13 isolates studied by WGS, all carried the *fosX* gene, which confers resistance to fosfomicin. Subsequently, the *lin* gene, associated with resistance to lincosamides, was identified in 12 different isolates, except isolate E. Additionally, the *norB* gene associated with resistance to fluoroquinolones was detected in five isolates (isolates A, B, F, H, and L). Additionally, *tetM*, detected in isolate L, confers resistance to tetracycline. The *qacJ* gene, known for conferring an efflux mechanism and resistance to disinfectants and antiseptics, was detected in two isolates (isolate H and isolate L).

Analysis of virulence factors identified 92 associated genes, and all the strains had the following 58 genes: *AgrA* (regulator of accessory gene regulator (agr) system), *FlaA* (encodes flagellin A, a component of flagella involved in motility), *FlgC* and *FlgE* (involved in flagellar assembly), *GadB* and *GadC* (part of acid resistance system), *Gmar* (glyoxalase/bleomycin resistance protein/dioxygenase), *OppA* (oligopeptide transport system substrate-binding protein), *OrfX* and *OrfZ* (proteins), *Rli55* and *Rli60* (RNAIII-inhibiting peptide, involved in RNA regulation), *Rsbv* (regulatory factor involved in stress response), *bilE* (resistance protein E), *bsh* and *btlB* (salt hydrolase), *codY* (global regulator of metabolism and virulence genes), *ctaP* (copper transporter ATPase), *dal* (D-alanine-D-alanine ligase, involved in cell wall biosynthesis), *degU* (transcriptional regulator, involved in biofilm formation and motility), *dltA* (D-alanyl-lipoteichoic acid biosynthesis protein, involved in cell wall modification), *fbpA* (ructose–bisphosphate aldolase, involved in glycolysis), *hly* (hemolysin), *inlC* (internalin C: cell invasion), *lgt* (galactosyl transferase, involved in lipoteichoic acid biosynthesis), *lhrC* (lipoprotein), *lipA* (lipoate synthase), *lisK*, *lisR, stp, tcsA, virR,* and *mogR* (virulence regulation), *lmo0610*, *lmo2085* and *uHp*t (proteins), *lpeA* and *lplA1* (lipoprotein), *lsp* (signal peptidase, involved in protein secretion), *mpl* (zinc–metalloprotease), *mprf* (phosphatidylglycerol lysyltransferase, involved in membrane composition), *per, pgdA* (deacetylates: cell wall modification), *pgla* (phospholipase), *plcA* (phosphadidylinositol phosphodiesterase (PI-PLC), *prfA* (regulation), *prsA*2 and *tig* (chaperone, involved in protein folding and secretion), *pycA* (pyruvate carboxylase, involved in metabolism), *recA* (recombinase A, involved in DNA repair and recombination), *relA* (involved in stress response), *secA2* (protein translocase subunit, involved in protein secretion), *sigB* (sigma factor B, involved in stress response and virulence gene regulation), *sipX* and *sipZ* (putative effector protein), sod (involved in oxidative stress response), *srtA* and *srtB* (involved in cell wall anchoring of surface proteins), and *svpA* (secreted virulence protein). Other virulence genes were also identified, such as *ActA* (*n* = 4), *Ami* (*n* = 8), *Aut* (*n* = 11), *Eut_operon* (*n* = 8), *lap* (*n* = 12), *lapB* (*n* = 8), *OatA* (*n* = 7), *chiA* (*n* = 12), *ClpB* (*n* = 3), *ClpC* (*n* = 4), *ClpE* (*n* = 11), *ClpP* (*n* = 12), ctsR (*n* = 12), *fri* (*n* = 7), *fur* (*n* = 12), *gtcA* (*n* = 12), *hfg* (*n* = 13), *hupC* (*n* = 12), *iap* (*n* = 5), *inlA* (*n* = 12), *inlB* (*n* = 7), *lmo0610* (*n* = 8), *murA* (*n* = 7), *plcB* (*n* = 7), *vip* (*n* = 6), *GadA* (*n* = 8), *htrA* (*n* = 9), *inlF* (*n* = 4), *inlH* (*n* = 6), *inlJ* (*n* = 5), *inlK* (*n* = 4), *lm0514* (*n* = 2), *lm2026* (*n* = 3), and *intA* (*n* = 7). 

### 2.5. Evaluation of the Biofilm Formation of L. monocytogenes Isolates

**Figure 2:**
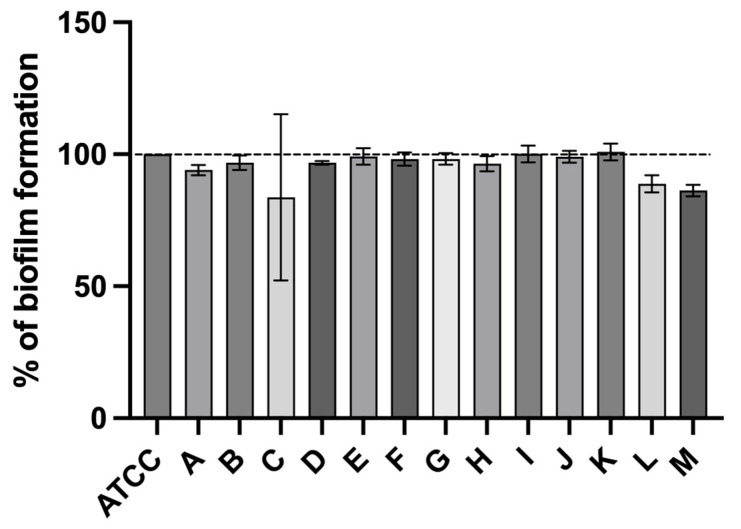
% Biofilm formation capacity (expressed as % in comparison to reference strain) of *L. monocytogenes* isolated from different food products and food-associated environments. Statistical significance was determined using Tukey’s multiple comparison test.

To ensure greater consistentcy in the comparison of the results, these were normalized against *L. monocytogenes* ATCC 7973. As shown in Figure 2, all strains were observed to have a high biofilm-forming capacity, with no significant differences between strains originating from food products and strains from food-related environments. Strain C, isolated from a turkey skewer with chorizo and chili peppers, had the lowest biofilm-forming ability, followed by strains L and M, obtained from a food production facility.

## 3. Discussion

Monitoring the prevalence, antimicrobial resistance, and genetic diversity of *L. monocytogenes* in foods is important for risk assessment, source identification, and establishment of control measures, as well as for building an effective surveillance system for this pathogen. The importance of *L. monocytogenes* for public health is emphasized by its frequent contamination of various food products [13,31]. When compared to global reports, there is high variability and different range of prevalence rates for *L. monocytogenes* in different countries. RTE food samples in Poland [32] had a higher prevalence of 13.5%, while semi-finished meat products in Russia [33] had 12% positivity. Turkey [34] and Estonia [35] had lower rates at 5% and 3.6%, respectively, and in Northern Greece [36], analysis of raw meat, raw meat preparations, ready-to-eat meat products, processing surfaces, and the environment showed a prevalence of 3.96%. When it comes to livestock, especially beef, pork and chicken, a study in China found an incidence of 7.1%, while in Europe the incidence was slightly higher at 8.3% [37]. Regional variations in food safety are influenced by a variety of factors such as differences in food safety regulations, surveillance intensity, and reporting practices. Different measures and the implementation of good agriculture practices can be applied in farming and food of animal production stages to prevent or reduce food safety hazards. The EU and the US have adopted preventive approaches to improve food quality assessment, including monitoring the food industry environmental microbiome [38], preventing cross-contamination in food processing, rotation disinfectants to reduce persister strains, and testing for the presence of *L. monocytogenes* regularly [39]. 

In our study, although *L. monocytogenes* isolates were found to be susceptible to a wide range of antibiotics, some strains showed to be susceptible to antibiotics such as meropenem, ampicillin, vancomycin, rifampicin, and ciprofloxacin. The highest resistance was to trimethoprim–sulfamethoxazole, to which 38.4% of the isolates were resistant, followed by erythromycin and meropenem, to which 15.5% and 7.6% of the isolates were resistant. *L. monocytogenes* has developed acquired natural resistance to a wide range of β-lactams and cephalosporins [40]. As such, based on the antimicrobial resistance profiles observed in the present study, the detection of reduced susceptibility to first-line antibiotics, such as penicillin, ampicillin, and trimethoprim sulfamethoxazole, is of concern. The resistance of *L. monocytogenes* to these antibiotics is likely due to the overuse of antibiotics in livestock for both growth promotion and treatment of bacterial infections [41]. 

This study also identified the presence of five resistance genes, including genes conferring resistance to fosfomycin (*fosX*), lincosamides (*lin*), fluoroquinolones (*norB*), and tetracycline (*tetM*). In addition, two isolates carried the *qacJ* gene, which is associated with the efflux mechanisms conferring resistance to disinfecting agents and antiseptics. This resistance complicates eradication efforts, especially in the food production environment. This two *L. monocytogenes* were isolated from different environments, such as slaughterhouse floor and catering establishments, which suggests that these genes are widely distributed in food and food environments. The evidence of sanitizer tolerance, resistance, and an enhanced ability to form biofilms suggests that we are in the presence of a strain classified as persistent. It has been documented that *L. monocytogenes* strains possess the capability to adapt to biocides, specifically ammonium quaternary compounds (referred to as quats or QACs), and a potential association between this adaptation and the development of resistance to the fluoroquinolone antibiotic ciprofloxacin has been suggested [42].

In recent years, *L. monocytogenes* has shown an increase in resistance to antibiotics, particularly in geographic regions with varied antimicrobial use [43]. While widespread resistance was not observed in our study, resistance to antibiotics like penicillin and trimethoprim–sulfamethoxazole was noted in food (sample A and sample B) and environmental samples (sample H, sample K, and sample L) over time. This highlights the potential for antibiotic efficacy loss in the future. The detection of multidrug-resistant strains in various food or food-related samples is a major concern worldwide, as showed in studies conducted in Spain [43] and South Africa [44]. In Spain, poultry preparation samples found that 49.1% of isolates exhibited an MDR phenotype, and in South Africa, resistance against various antibiotics was also reported, namely against sulfamethoxazole, trimethoprim, and erythromycin. Regarding the presence of multidrug-resistant strains, 38.10% were found to have an MDR phenotype [44]. Similarly, a study in China [41] found low resistance rates in isolates from food, livestock, and clinical samples, with tetracycline showing the highest resistance. The high prevalence of antimicrobial resistance observed in these studies may be strongly related to the widespread use of these drugs in veterinary medicine. Therefore, MDRs typically pose challenges in effectively treating the infections they cause, which can lead to higher hospital costs and prolonged antibiotic treatment [43,44]. 

Regarding *L. monocytogenes* typing, we found considerable diversity as eight STs were identified, ST9, ST155, ST3, ST121, ST8, ST87, ST1, and ST3207, with ST3 and ST121 being the most common. Moreover, the MDR isolate belonged to ST87. Notably, we detected a cluster of three very closely related ST3 isolates from 2014, suggesting a common contamination source. A common food denominator of these three samples is “alheira”, which is a traditional smoked naturally fermented meat sausage produced in the north of Portugal. This observation suggests that outbreaks are often linked to contamination from processing and handling environments and sanitation failures [45]. External factors like poor hygiene practices and ineffective disinfectants contribute to continued exposure. Specific genes in certain *L. monocytogenes* strains contribute to their persistence. Addressing residual strains is a major challenge for food manufacturers, as they can cause cross-contamination and be difficult or impossible to remove [7]. In the Czech Republic, ST3 is prevalent in RTE foods and is a dominant clone globally, found in both human cases and food sources [46]. Studies in Brazil [47] and Poland [48] also found ST3 in environmental samples, cheese products, and RTE foods. Our results are consistent with results obtained in other studies. The ST121 strain was found in food products in 2014 and food establishments in 2018, highlighting its prevalence and distribution in different geographical regions. A study in China [41] showed ST121 in food isolates, indicating its presence in that region. In Switzerland [49], studies revealed that within the food isolate category, ST121 had a lower incidence of human listeriosis. This suggests that ST121 is common in food-related contexts but less harmful clinically [41]. CC121 clonal complexes are often associated with food origins and are characterized by hypovirulence and increased susceptibility to infection in people with severe immunodeficiency diseases. Also, CC121 was observed to persist in food production environments. A study by Maury et al., involving 6641 CC121 food isolates from 2005–2016, found that 4.4% of the samples originated from dairy products, 53.2% were meat-related, and 21.2% were seafood-related based on 6641 isolates [50]. In our study, the presence of ST121 was detected in both food and environmental samples over different time periods. CC121 strains were found in a catering establishment sample (sample K) in 2018 and a slaughterhouse floor sample (sample H) in 2019. In 2021, meat preparation sample (sample A) showed a unique ST designated 3207 belonging to CC121. ST9 [50], which is commonly found in food, was also found in two samples. Studies in China [51] showed that ST9 is more prevalent in food isolates, suggesting an association with food and processing environments. In Latvia [52], certain sequence types including ST9 are frequently identified, with associations to food, food-processing environments, and ruminant farms. Various studies have found ST8, ST87, and ST1 in food preparation environments, particularly in ready-to-eat foods [53]. ST8 is common and known to be persistent in food samples [41,54], linked to foodborne listeriosis outbreaks [41]. Between May and September 2017, ST8 was associated with the consumption of ready-to-eat cold-smoked salmon produced in Poland. This multi-country outbreak of 12 cases of listeriosis caused by *L. monocytogenes* ST8 was reported in three EU/EEA countries: in Denmark (six cases), Germany (five cases) and France (one case) [55]. Our ST8 *L. monocytogenes* isolate was obtained in 2018 from catering establishments; it belongs to CC8 and lineage II. It has been previously shown that *L. monocytogenes* ST8 strains demonstrate strong viability in food processing plants, with potential for transfer between different food businesses via raw food materials. This phenomenon may contribute to the persistent presence of specific clones of *L. monocytogenes* in environmental niches and facilitate their transmission between food establishments [53]. ST1 and ST87 have been detected in food products, such as raw milk cheeses in Portugal [56] and South Africa [57]. Another study conducted in Switzerland [49] found ST1 to be the most prevalent sequence type in human strains. The diverse sequence types of *L. monocytogenes* identified in our study underscore the dynamic nature of strains in various environments and highlight the diverse roles and associations of these sequence types in food safety and public health. 

All the isolates harbored adherence and invasive genes LIPI-1 (*Listeria* Pathogenicity Island 1), LIPI-2 (*Listeria* Pathogenicity Island 2), and LIPI-3 (Listeria Pathogenicity Island 3), and *L. monocytogenes* virulence is directly related to invasiveness and its capability to multiply in a wide range of eukaryotic cells. The presence and prevalence of virulence genes can reflect the risk levels of different *L. monocytogenes* strains, and it is suggested that persistent strains may be better adapted to grow under stressful conditions, such as temperature, NaCl concentration, and acidity [58]. The utilization of WGS has emerged as a promising and predictive method for assessing the virulence potential and functional characteristics of virulence factors and aids in comprehending virulence mechanisms and strategies but also holds the potential to monitor the risk of listeriosis outbreaks [59]. The significant presence of virulence factors observed in our study correlates with the biofilm formation capacity of the analyzed strains. This connection is evident through the detection of virulence factors associated with biofilm formation, suggesting that the strains can be classified as persistent.

Biofilms are aggregates of microbial cells that are interconnected and adhere tightly to each other or to a surface. Encased in an extracellular multicellular matrix, they play an important role in microbial survival in harsh environments by providing phenotypic flexibility and ecological benefits [60]. *L. monocytogenes* is resilient and can colonize and survive in food processing facilities. The ability to form biofilms promotes its growth and proliferation in harsh environments [44,61]. In our study, the evaluation of 13 strains of *L. monocytogenes* from food products and environments related to food production demonstrated the potential to form biofilms. All strains had a strong biofilm-forming ability, and there were no significant differences between strains obtained from food products and strains from environments associated with food production. The high production of biofilms observed in *L. monocytogenes* strains may also be due to the presence of the *DegU* virulence gene, which plays a role in biofilm formation and is associated with biofilm formation. Several studies have evaluated biofilm formation. In one of them, samples of food products were used [44], including milk and milk products, and in another study, samples were obtained from the environment [61]. In these studies, 95.2% of the strains isolated from milk and 68.4% of the strains isolated from environment samples showed the ability to form biofilm [44,61]. 

## 4. Materials and Methods

### 4.1. Sample Collection and Processing

A total of 227 samples obtained from food products (meat preparation, cheeses, and raw milk) and food-associated environments (slaughterhouse floor and catering establishments) were collected from 2014 to 2022. Ten grams per sample were used, and all the samples were collected aseptically and diluted with 90 mL of sterile buffered peptone water (0.1% *w*/*v*) and homogenized in a stomacher (Lab Blender, West Sussex, UK) for 30 s at room temperature. Further dilutions (1:10) were obtained in sterile peptone water (0.1% *w*/*v*) (BPW, Biokar diagnostics, Allonne, France) and inoculated in triplicate [62]. 

### 4.2. Isolation and Identification of L. monocytogenes

According to ISO 11290-1 [63], half Fraser broth (VWR Chemicals, Radnor, PA, USA) was used as the main enrichment medium, followed by incubation at 30 °C for 24 h. Then, 0.1 mL of the aliquots were transferred to Fraser broth (VWR Chemicals) and incubated at 37 °C for 48 h. Cultures obtained from half Fraser broth/Fraser broth were then subjected to further analysis: 0.1 mL aliquots were placed on supplemented Oxford agar (VWR Chemicals) and supplemented Palcam agar (VWR Chemicals), incubated at 37 °C for 24–48 h. To confirm the colonies, a series of tests including catalase reaction, Gram-stain, hemolysis assessment, and carbohydrate utilization were performed according to ISO 11290-1 [64]

### 4.3. Taxonomic Identification of Bacterial Isolates

Total bacterial DNA from the 13 presumptive *L. monocytogenes* isolates was extracted using the InstaGene Matrix (BioRad Laboratories, Inc., Hercules, CA, USA) according to manufacturer’s instruction. The *16S rDNA* gene was amplified by PCR and then sequenced. PCR amplifications were performed using 25 μL of DreamTaq Hot Start PCR Master Mix 2x (Thermo Scientific, Waltham, MA, USA), 0.5 μM fD1 (5′-AGAGAGTTTGATCCTGGCTCAG-3′), 0.5 μM rD2 (5′-TAAGGAGGAGGTGATCCAGCC-3′), 50–100 ng of purified DNA and 19 μL of molecular biology-grade water (Thermo Scientific). PCR mixtures were subjected to several amplification cycles, starting with an initial denaturation cycle (95 °C, 3 min), followed by 35 cycles of denaturation (95 °C, 30 s), hybridization (60 °C, 30 s), elongation (72 °C, 1 min), and ending with a final elongation cycle (72 °C, 5 min) in a thermal cycler (Eppendorf, Hamburg, Germany). The resulting amplicons were then purified using the Nucleospin Gel and PCR Clean-up kit (Macherey-Nagel^TM^, Düren, Germany) and sent to Eurofins Genomics (Ebersberg, Germany) for DNA sequencing. To determine their taxonomic identification, the nucleotide sequences were analyzed using the BLAST nucleotide server of the National Center for Biotechnology Information (NCBI) (https://blast.ncbi.nlm.nih.gov/, accessed on 28 February 2024).

### 4.4. Antimicrobial Susceptibility Testing

Antibiotic susceptibility testing was conducted using the Kirby–Bauer disk diffusion method as recommended by the European Committee on Antimicrobial Susceptibility Testing (EUCAST). Briefly, 5–6 overnight colonies were suspended in 1 mL of a 0.9% NaCl solution, and the turbidity of this suspension was adjusted to a 0.5 McFarland standard, corresponding approximately to 1–2 × 10^8^ CFU/mL. The suspension was used to inoculate Mueller–Hinton blood agar. The isolates were tested against a panel of twelve antimicrobial agents with relevance to human and animal health. The diameter of the zones exhibiting complete inhibition was measured. The following panel of antimicrobial disks and concentrations was used: meropenem (MRP-10 μg), ampicillin (AMP-10 μg), ciprofloxacin (CIP-5 μg), clindamycin (CD-2 μg), erythromycin (E-15 μg), kanamycin (KAN-30 μg), gentamicin (CN-10 μg), penicillin G (PEN-10 IU), rifampicin, vancomycin (VA-30 μg), trimethroprim-sulphamethoxazol (SXT-1.25 μg/23.75 μg), and linezolin (LZ-10 μg) [65,66]. Data interpretation was performed according to the recommendation of EUCAST guidelines; in cases where the EUCAST guidelines lacked resistance criteria for *Listeria*, the guidelines followed were those recommended for *Staphylococcus aureus* and *Enterococcus* spp. by CLSI guidelines [15]. 

### 4.5. L. monocytogenes Whole-Genome Sequencing

Genomic DNA was extracted from fresh cultures of all *L. monocytogenes* using the ISOLATE II Genomic DNA Kit (Bioline, London, UK) and quantified in the Qubit fluorometer (Invitrogen, Waltham, MA, USA) with the dsDNA HS Assay Kit (Thermo Fisher Scientific, Waltham, MA, USA) according to the manufacturer’s instructions. DNA was then prepared using the NexteraXT library preparation protocol (Illumina, San Diego, CA, USA), and then cluster generation and sequencing (2 × 150 bp) on a NextSeq 2000 instrument (Illumina) were performed. Quality control, trimming, and de novo genome assembly were performed with the INNUca pipeline v4.2.2 “https://github.com/BUMMI/INNUca”, accessed on 28 February 2024 [67] using default parameters. In brief, FastQC v0.11.5 “http://www.bioinformatics.babraham.ac.uk/projects/fastqc/”, accessed on 28 February 2024 and Trimmomatic v0.38 [68] were used for reads of quality control and improvement. De novo genome assembly was performed with SPAdes v3.14 [69]; reads were aligned with Bowtie v2.2.9 [70] and the assembly was polished with Pilon v1.23 [71] as integrated in INNUca v4.2.2. Species confirmation/contamination screening was performed with Kraken2 v2.0.7 [72]. ST determination was performed with mlst v2.18.1 “https://github.com/tseemann/mlst”, accessed on 28 February 2024. To assess the genomes for acquired antibiotic resistance genes and VFs, ResFinder and VirulenceFinder v2.0 (Center for Genomic Epidemiology, Technical University of Denmark, Lyngby, Denmark) servers were used. The Comprehensive Antibiotic Resistance Database (CARD) was used to search the genome for acquired antibiotic resistance genes.

ResFinder and VirulenceFinder was firstly employed with cut-offs of 95% percentage of identity (ID) and 60% for the minimum length of coverage. The genome sequences were aligned against the protein sequences from ARG using the default parameters and the Perfect, Strict and Loose hits criteria from the Comprehensive Antibiotic Resistance Database (CARD), only hits with >70% ID were retained for analysis.

### 4.6. Core-Genome Clustering Analysis of L. monocytogenes Isolates

For *L. monocytogenes* strains, allele calling was performed on polished genome assemblies with chewBBACA v2.8.5 [73] using the core-genome Multi Locus Sequence Typing (cgMLST) Pasteur schema built from the 1748 loci [27] available at the Chewie-NS website “https://chewbbaca.online”, accessed on 28 February 2024 [74]. cgMLST clustering analysis was performed with ReporTree v.2.0.3, available via this GitHub repository: “https://github.com/insapathogenomics/ReporTree”, accessed on 28 February 2024 [28] using GrapeTree (MSTreeV2 method) [29], with clusters of closely related isolates identified and characterized at a distance thresholds of 1, 4, 7, and 15 allelic differences (ADs). A threshold of seven ADs may provide a proxy for identifying genetic clusters with potential epidemiological concordance (i.e., “outbreaks”) [30]. Interactive phylogenetic tree visualization was conducted with GrapeTree [29].

### 4.7. Biofilm Formation Assay

The biofilm formation assay was conducted according to a previously described protocol [75]. In brief, two colonies from a fresh culture were transferred into tubes containing 3 mL of Tryptic Soy Broth (TSB, Oxoid, Basingstoke, UK) and incubated at 37 °C for approximately 16 ± 1 h with continuous shaking at 120 rpm, utilizing the ES-80 Shaker-incubator from Grant Instruments (Cambridge, UK). After this incubation period, the bacterial suspension was adjusted to an optical density equivalent to 1 × 10^6^ colony-forming units. Subsequently, 200 μL of bacterial suspensions from different isolates were added to individual wells of a 96-well microplate. A positive control, *Listeria* ATCC 7973, was included in all plates, and a fresh uninoculated medium was used as a negative control. The plates were then incubated at 37 °C for 24 h without shaking. Each experiment was performed with seven replicates and performed three times. Biofilm volume assessment was performed using Crystal Violet (CV) staining, following the procedure described by Peeters et al. (2008) [76] with some modifications. After the incubation period, the plates were washed twice with 200 μL of distilled water to remove non-adherent bacterial cells. The plates were then air-dried at room temperature for approximately 2 h to fix the microbial biofilm; then, 100 μL of methanol (VWR International Carnaxide, Portugal) was added to each well and incubated for 15 min. Then, the methanol was removed, and the plates were air-dried again at room temperature for 10 min. To dissolve CV, 100 µL of 33% (*v*/*v*) acetic acid was added, and absorbance was measured at 570 nm using a microplate reader (Bio Tek elX808U, Winooski, VT, USA) [75]. Biofilm formation results for each isolate were presented as a percentage of the results obtained for the reference strain.

### 4.8. Statistical Analysis

Descriptive statistics including mean (M) and standard deviation (SD) were used where appropriate. Skewness and kurtosis coefficients were calculated to assess univariate normality. The relationship between biofilm formation in different samples from different sources was analyzed using one-way analysis of variance (ANOVA) followed by Tukey’s post-hoc test and independent samples *t*-test. All statistical analyzes were performed using SPSS (IBM SPSS Statistics 26), and statistical significance was set at *p* < 0.05.

## 5. Conclusions

The growing threat of antimicrobial resistance in *L. monocytogenes* is of concern and might pose significant challenges in effectively treating listeriosis in the future, highlighting the need for systematic and inter-sectorial surveillance strategies. Analysis of antimicrobial resistance revealed resistance to important antibiotics, including penicillin, ampicillin, and trimethoprim–sulfamethoxazole, commonly used as a first-line treatment for listeriosis. In this study, we identified different STs of *L. monocytogenes* strains and characterized their dynamic properties in different environments. The identification of STs such as ST8, ST87, and ST1 in food preparation environments and the presence of STs in foods highlights the potential for cross-contamination and persistence within the food processing and handling continuum. These STs play various roles in food safety and the presence of STs in food and food-processing environments. Educating consumers, food handlers, and food industry professionals about proper handling practices, hygiene protocols, and the consequences of antimicrobial overuse can contribute to safer food production and consumption practices. Comprehensive surveillance is essential to understand the dynamic nature of antimicrobial resistance in *L. monocytogenes* obtained from food products and food-associated processing environments and to develop strategies to minimize the potential public health risks associated with antibiotic-resistant *L. monocytogenes* and adopting integrated food safety management systems.

## Figures and Tables

**Table 1:** Antimicrobial resistance profiles from *L. monocytogenes* strains and sequence types identified in *L. monocytogenes* isolates.

Strain	Isolation Year	Source	Antibiotics Tested		Multi-Resistance Profile	Sequence Type	cgMLST	Clonal Complex	Lineage
			Resistant	Susceptible					
A	2021	Meat preparation	STX	MRP-AMP-CIP-DA-E-K-CN-P-RD-VA-LNZ	No	ST3207		CC121	II
B	2022	Alheira	DA-SXT	MRP-AMP-CIP-E-K-CN-P-RD-VA-LNZ	No	ST9		CC9	II
C	2022	Turkey kebab with chorizo and peppers	P	MRP-AMP-CIP-DA-E-K-CN-P-RD-VA-LNZ-SXT	No	ST155		CC155	II
D	2014	Alheira pasta packaged in aerobiosis	P	MRP-AMP-CIP-DA-E-K-CN-P-RD-VA-LNZ-SXT	No	ST3	cluster_1 (≤7 ADs)	CC3	I
E	2014	Alheira dough in vacuum	-	MRP-AMP-CIP-DA-E-K-CN-P-RD-VA-LNZ-SXT	No	ST9		CC9	II
F	2014	Alheira pasta packaged in aerobiosis	P	MRP-AMP-CIP-DA-E-K-CN-P-RD-VA-LNZ-SXT	No	ST3	cluster_1 (≤7 ADs)	CC3	I
G	2014	Fresh alheira pasta	-	MRP-AMP-CIP-DA-E-K-CN-P-RD-VA-LNZ-SXT	No	ST3	cluster_1 (≤7 ADs)	CC3	I
H	2019	Slaughterhouse floor	E-SXT	MRP-AMP-CIP-DA- K-CN-P-RD-VA-LNZ	No	ST121		CC121	II
I	2018	Catering establishments	-	MRP-AMP-CIP-DA-E-K-CN-P-	No	ST8		CC8	II
J	2018	Catering establishments	-	MRP-AMP-CIP-DA-E-K-CN-P-RD-VA-LNZ-SXT	No	ST3		CC3	I
K	2018	Catering establishments	MRP-DA-E-CN-RD-VA-SXT-LNZ	AMP-CIP-K-P	Yes	ST87		CC87	I
L	2018	Catering establishments	SXT	MRP-AMP-CIP-DA-E-K-CN-P-RD-VA-LNZ	No	ST121		CC121	II
M	2018	Catering establishments	-	MRP-AMP-CIP-DA-E-K-CN-P-RD-VA-LNZ-SXT	No	ST1		CC1	I

Legend: cgMLST—core-genome clustering analysis; ST—sequence type; MRP—meropenem; AMP—ampicillin; CIP—ciprofloxacin; DA—clindamycin; E—erythromycin; K—kanamycin; CN—gentamicin; P—penicillin G; VA—vancomycin; SXT—trimethoprim–sulfamethoxazole; LNZ—linezolin.

## Data Availability

Sequencing data generated within this study are deposited in the European Nucleotide Archive (ENA) database under the ENAProject number PRJEB31216.
